# The Treatment of Women in Confinement—Proceedings of I. H. A.

**Published:** 1891-02

**Authors:** 


### THE TREATMENT OF WOMEN IN CONFINEMENT.

(Proceedings of I. H. A. Morning Session. June 25th, 1890.)

Dr. Custis read a paper entitled, "The Homœopathic Obstetrician," which excited the following discussion :

Dr. J. B. Bell—I wish to enter a strong protest against either Cosmoline or Lanolin coming in contact with a human being. They produce symptoms, and are therefore not entirely innocuous. And I want to protest still more strongly against Chloroform.

To give Chloroform requires the entire attention of a skillful man. It is not less dangerous to a lying-in woman than to others, and therefore the obstetrician should not trust himself to give it while his attention is taken up with other matters. Besides, it has a tendency to cause fatty degeneration, even from a few applications. Ether, on the other hand, given in the small quantity required, does not call for the services of another, does not vomit, and does not prostrate.

Dr. Hawley—What is the use of Cosmoline or of Lanolin, or of anything of that kind ? Oils and fats are always heating to a mucous surface. I once took about four ounces of lard out of the vagina of a woman in labor. It had been put there by the doctor to lubricate the parts. Twenty-four hours had passed without any progress being made, and beneath that lard I found the vagina just as dry as a bone.

The baby was born in fifteen minutes afterward.

Dr. H. C. Allen—I should like to know why Dr. Custis always gives Pulsatilla in the last few weeks of gestation, without any indications ?

Dr. Custis—Because, since I have been doing it I have never had a case of abnormal presentation, and because I have never seen any trouble arising from it.

Dr. Reed, in answer to a question by Dr. Alice Campbell, said : I am opposed to the administration of any remedy as a preparation for labor, unless it is indicated. To give Pulsatilla simply because it *is* Pulsatilla, and is often indicated, is not Homœopathy. You are liable to have just as many Nux-vomica or Hyoscyamus conditions as of Pulsatilla. Pulsatilla should be given only when it is indicated. If gestation is normal then any remedy will do harm. There is no more excuse for giving Pulsatilla than there is for giving Chamomilla or Gelsemium, or any other remedy at such a time.

Dr. Reed then announced himself as also opposed to the use of any of the greases. He thought Chloroform might sometimes be useful, or, better still, Ether.

Dr. Carleton—A protest to a protest. Anything in this world but Ether. There may have been more deaths from Chloroform than from Ether, but there have also been deaths from Ether in the hands of the best men. Even with a careful administration of Ether it is often impossible to revive the patient without the bastinado. I never intrust the administration of any anæsthetic to the nurse ; there is too much danger of death.

Dr. Farley—In the paper read, Merc-cor. is advised if there should be albumen in the urine. I do not think that advice should go unchallenged.

Dr. Reed—I entirely disagree with Dr. Custis on the Merc-cor. question. Never give any medicine unless it is indicated, and I do not think that albumen in the urine without any subjective symptoms is an indication for Merc-cor. In regard to an assistant in labor you should remember that many babies are born in the country, ten miles from any help, so that an assistant is not so easy to get. I suppose I have performed instrumental deliveries twenty-five times without help.

Dr. Hitchcock—A gentleman of large experience told me that he had never had to use forceps. Are forceps a necessity ?

Dr. Wesselhœft—Dr. Bell once left a young man in charge of his practice during a short absence. The young man had two cases of placenta previa before Dr. Bell got back. Dr. Bell had never up to that time seen a case of placenta previa.

I left the same young man in charge of my practice and before I got back he had a placenta previa. I have never seen a case of that kind. Now that young man must have been unlucky.

Now when a man says he has gone through a long life of medical practice without using the forceps, that is not an argument. He must have been awfully lucky, that's all. I have been obliged to use the instruments after the most careful and thoughtful administration of remedies which helped nothing. My father, who practiced medicine for forty years, had very remarkable mechanical skill. He was a whitesmith by trade and made all his own instruments. In forty years he had only used instruments in labor three times. I have used them much oftener than that.

Dr. Carr—The forceps are certainly necessary at times, but in the majority of abnormal cases we can get a natural delivery by the use of remedies, and hence I object to a too early or thoughtless use of instruments. Their proper use may sometimes prevent serious laceration of the perineum. They may even save life and so should always be on hand. I have had two cases of placenta previa. I have used Chloroform but never Ether.

I think also there is too much haste in cutting the cord and the rule to wait until it ceases to pulsate, as given by Dr. Custis, is correct, but I do not think any remedy should be given during gestation unless indicated.

Dr. Kent—All through the history of obstetrics we find women have died during child-birth, and I have no doubt but that many more women would die in the present time were it not for the forceps. The indicated remedy may not be easily found at the time, and not everybody is expert enough to find a remedy to correct the wrong in advance. I have had to deliver with forceps on that account. Yet I have known men with many times the obstetrical practice of mine who have never used the forceps.

It is a difficult question to solve concerning anæsthetics. I remember one case in which I expected to deliver with forceps without an anæsthetic as I had done before and have done since. It was a case in which thickening and infiltration of tissue had followed a pelvic cellulitis. The cervix was undilatable. Dilatation had to be performed mechanically and the forceps had to be introduced high. The intense agony, the extreme suffering it seemed to me would destroy the woman's life. It was a rare case, and I had to give Chloroform, at the same time I do not want to be understood as indorsing or advising the use of Chloroform. But there are conditions which remove the case from the realm of medicine to that of surgery. Then it becomes a necessity to use Chloroform.

Dr. Custis—I am glad that my paper has been so successful in eliciting so interesting a discussion. I do not see how the remedies can possibly do away with the forceps, where we have non-conformity between the axes of the head and of the pelvis. If the head is too large it is beyond the province of remedies to decrease its size. The forceps must then be used. I have used Chloroform only in obstetrical practice. The stimulus to the heart of child-bearing seems to counteract the depressing effect of the Chloroform and I have never had any bad results. It is, of course, best to have another physician present, but not absolutely necessary. I have elsewhere, in a paper on Albuminuria, given the effect of Merc-cor. It produces an absolute physiological action in reducing the amount of albumen in the urine. Following this experience I have given Merc-cor. on a pathological basis on this symptom alone.

Dr. Hoyne—Did Dr. Custis ever hear of a death from the administration of Chloroform during labor?

Dr. Custus—No, sir, I never did.

